# Perspectives of Black and Hispanic Children Living in Under-Resourced Communities on Meal Preparation and Grocery Shopping Behaviors: Implications for Nutrition Education

**DOI:** 10.3390/ijerph182212199

**Published:** 2021-11-20

**Authors:** Chishinga Callender, Denisse Velazquez, Meheret Adera, Jayna M. Dave, Norma Olvera, Tzuan A. Chen, Shana Alford, Debbe Thompson

**Affiliations:** 1 USDA/ARS Children’s Nutrition Research Center, Department of Pediatrics, Baylor College of Medicine, 1100 Bates Street, Houston, TX 77030, USA; Chishinga.Callender@bcm.edu (C.C.); himedenisse@gmail.com (D.V.); msa8@rice.edu (M.A.); jmdave@bcm.edu (J.M.D.); 2 Psychological, Health and Learning Sciences Department, University of Houston, 3657 Cullen Boulevard Room 491, Houston, TX 77204, USA; nolvera@central.uh.edu (N.O.); tchen3@central.uh.edu (T.A.C.); 3 Health Research Institute, University of Houston, 4849 Calhoun Road, Houston, TX 77204, USA; 4 Common Threads, 222 W. Merchandise Mart Plaza, Suite 1212, Chicago, IL 60654, USA; salford@commonthreads.org

**Keywords:** minority, children, nutrition, meal preparation, grocery shopping, Black/African American, Hispanic, under-resourced, qualitative

## Abstract

Minority children living in under-resourced communities are at the greatest risk for obesity and poor diet quality. Child involvement in meal preparation may be a helpful strategy to improve diet quality. This paper explores minority children’s perspectives regarding this. Eighteen children participated in a mixed methods study (online surveys, telephone interviews). Descriptive statistics were calculated for child demographic and psychosocial factors. Thematic analysis was used to code and analyze the interviews. Most children reported having cooking experience (83%) and cooking with family (94%) and exhibited high cooking self-efficacy (21.8 ± 2.9) and positive cooking attitudes (25.7 ± 4.4). Children reported helping with meal preparation (50%) and grocery shopping (41%) sometimes. The qualitative data further supported the results obtained from the children’s psychosocial factors. Most children noted the importance of learning to cook with an emphasis on life skills. Children also shared their level of involvement in cooking and grocery shopping. Most children reported using technology when cooking to find demonstration videos and recipes. These findings highlight that minority children participate in meal preparation and grocery shopping. Their perspectives are important for the development of nutrition education programs to achieve equitable dietary outcomes in minority families living in under-resourced communities.

## 1. Introduction

Child obesity prevention is a critical public health issue in the United States, with approximately 19.3% of 2–19-year-old children being obese in 2017–2018 [1]. Obese children are at a greater risk for comorbidities such as type 2 diabetes mellitus and hypertension [2]. Racial and ethnic disparities exist in child obesity rates: the prevalence of obesity is highest in Hispanic (25.6%) and Black/African American (24.2%) children compared to non-Hispanic white (16.1%) children [1,3,4]. The prevalence of obesity is also higher among children living in under-resourced households [5]. Exploring effective ways to address the prevalence of child obesity and risks of comorbidities is an important step forward in reducing health disparities [6].

Although obesity is a multifactorial phenomenon, dietary intake through increased caloric intake is the most important factor contributing to obesity risk [2,7]. The home food environment [8], family meals [9], food preferences [10], and household income [11] contribute to children’s dietary behaviors. Children can also influence food behaviors in both themselves [9] and their family [12]. The diet quality of children in the United States is below the national dietary recommendations [13]. Furthermore, the School Nutrient Dietary Assessment found that the majority of children (88%) ate low-nutrient, energy-dense foods over a 24 h period [14]. Diet-related disparities for children and families are well-documented, with racial and ethnic minorities (i.e., Black/African American, Hispanic) reporting poorer diet quality compared to their non-Hispanic white peers [15,16]. These disparities also exist in low-income communities, as poor diet quality is associated with socioeconomic status [17].

Child involvement in home meal preparation may be a helpful strategy to improve diet quality in children [18]. Previous research revealed that child and adolescent involvement in meal preparation improved diet quality, including higher intakes of fruits and vegetables [18,19,20,21,22] and nutrients [22]. For young adults, frequent meal preparation has also been associated with improved diet quality, including eating five servings of fruits and vegetables daily and less consumption of fast foods [23].

Child and adolescent involvement in meal preparation has also been associated with positive psychosocial factors among children. A study conducted with fifth graders found an association between meal preparation and higher self-efficacy for making healthy food choices and higher fruit and vegetable preferences [24]. Another study with sixth to eighth graders found that meal preparation frequency was positively associated with cooking self-efficacy and food preparation techniques [25]. In addition, positive attitudes and higher self-efficacy towards healthy meal preparation were associated with children practicing healthy meal preparation [26].

Evidence suggests that encouraging child involvement in meal preparation may improve dietary intake and psychosocial factors related to diet and cooking. A cooking and nutrition education program for children from minority (i.e., Black and Hispanic), low-income families, found that the program increased fruit and vegetable consumption, cooking self-efficacy, nutrition knowledge, and the frequency of the child cooking at home [27]. The Cooking with Kids program with Hispanic children from low-income families found that cooking self-efficacy and attitudes towards cooking and fruit and vegetable preferences increased among students without cooking experience [28]. Similarly, this program found that vegetable preferences, self-efficacy, and attitudes toward cooking increased among non-Hispanic white students [29]. Few studies have qualitatively explored the perspectives of children regarding involvement in meal preparation [30,31] and grocery shopping behaviors. Given the potential of children being involved in food preparation to improve their diet-related behaviors, the perspectives of children from minority and low-income communities are essential for developing effective, acceptable, and equitable nutrition and cooking education programs. Thus, the purpose of this research was to explore perspectives regarding meal preparation and grocery shopping behaviors of minority children living in under-resourced communities.

## 2. Methods

This is a secondary data analysis of a larger mixed methods study conducted with minority parents and children living in under-resourced communities that explored their thoughts, experiences, and recommendations regarding nutrition education and cooking education programs. This paper addressed the following research questions: (1) What are children’s beliefs about meal preparation and grocery shopping? (2) What is a child’s level of involvement in meal preparation and grocery shopping?

### 2.1. Design

A mixed methods (surveys and interviews) design was used in the larger study. The protocol was approved the by the institutional review board at Baylor College of Medicine (H-44683).

### 2.2. Study Participants

Parent/child dyads were stratified by race/ethnicity (Black/African American, Hispanic) based on the parents’ self-identified race and/or ethnicity. Inclusionary criteria for the children included: being 8–13 years old, being fluent in English or Spanish, eligibility for free or reduced-price lunch at school, being healthy (i.e., no physical, health, or medical condition that would affect diet or participation in data collection), willingness to participate in an audio-recorded telephone interview, living in the same household as a parent, and willingness of parents to participate in study activities with the child. Exclusionary criteria included unwillingness to have the telephone interview recorded and to take photos for the study.

### 2.3. Recruitment

Recruitment began in early May 2019 and ended in mid-August 2019. Families living in under-resourced communities in the Houston metropolitan area were recruited by contacting them from the USDA/ARS Children’s Nutrition Research Center’s (CNRC) volunteer database and using referrals from recruited families. Families provided written informed consent prior to participation in the study. A more detailed description of the recruitment procedures is reported elsewhere [12].

### 2.4. Data Collection

Data collection began in June 2019 and ended in October 2019. Each child completed an online survey followed by a telephone interview. Surveys were hosted on a secure, password-protected server. Trained research coordinators conducted telephone interviews using a semi-structured script. Separate scripts guided parent and child interviews. This paper includes quantitative data from the children’s questionnaires on the following psychosocial measures: cooking self-efficacy, cooking attitude, cooking experience, and involvement in grocery shopping/food preparation. Cooking self-efficacy, cooking attitude, and cooking experience measures were adapted from valid and reliable measures developed for the Cooking with Kids Program [29,32]. Cooking self-efficacy was measured using a three-point scale (yes, no, not sure) consisting of eight items (e.g., I can make a snack with fruit, I can help make a family meal, I can cut up food). Possible self-efficacy scores ranged from 8 to 24, with higher scores indicating greater self-efficacy. Cooking attitude was measured using a five-point scale (really like, kind of like, don’t like, really don’t like, not sure) consisting of six items (e.g., feelings towards cooking, measuring ingredients, making snacks). Possible cooking attitude scores ranged from 6 to 30, with higher scores indicating more positive attitudes. Cooking experience was measured using a two-point scale (yes, no) consisting of three items (i.e., I have cooked before, I have cooked with family/friends). Involvement in grocery shopping and food preparation was measured using a four-point scale (never, sometimes, often, always) consisting of two items (e.g., How often do you shop or help shop for groceries? How often do you help make food for your meals?). This paper also includes children’s demographic characteristics from the online survey completed by parents.

In addition to the survey data, qualitative data from the children’s interview questions are included in this paper. Sample interview questions included, “What does the word cooking mean to you?” “On a scale of 1 (not confident), 2 (not sure), 3 (confident), how confident are you in your cooking skills?” “Do you cook meals with your parents?” “Do you go grocery shopping with your parents?” Probes and prompts were used to clarify and expand responses. Each interview was digitally recorded, transcribed, and reviewed for accuracy prior to analysis by trained research staff.

### 2.5. Data Analysis

#### 2.5.1. Surveys

Descriptive statistics were calculated for child demographic and psychosocial variables. Means and standard deviations were calculated for the psychosocial variables (cooking self-efficacy, cooking attitude). Frequencies and percentages were calculated for the demographic characteristics and the following psychosocial variables: cooking experience and involvement in grocery shopping/food preparation. All statistical analyses were conducted using SAS (version 9.4, SAS Institute Inc., Cary, NC, USA, December 2010).

#### 2.5.2. Interviews

Two independent coders used thematic analysis to code and analyze the interview transcripts [33]. A priori codes were initially used to code the data. During the coding process, emergent codes were added to extensively capture the perspectives of the children. Analysis was conducted on English language transcripts (i.e., English language transcripts and translated Spanish language transcripts). Findings from child interview transcripts were organized by interview questions on meal preparation and grocery shopping. A codebook was maintained and routinely updated to reflect new codes, definitions, and key decisions. Interview questions with yes and no responses were summed to indicate the number of participants who provided each response. The survey results were backed by related qualitative findings. Verbatim quotes were used to support qualitative findings, and quotes were identified by the following ethnic categories: A = African American/Black and H = Hispanic. To help differentiate the quotes, a number (from 1 to 18) was assigned to each child.

## 3. Results

### 3.1. Participant Characteristics

Eighteen children enrolled in the study and completed all data collection activities. Over half of the children participating in the study were female (56%) and Black/African American (56%). Half of the children (50%) participating in the study were identified as Hispanic by their parents. The majority of the children were in the age range of 11–13 years old (78%), while a few were in the age range of 8–10 years old (22%). All children recruited for the study met the inclusionary criteria.

### 3.2. Children’s Psychosocial Variables and Meal Preparation and Grocery Shopping Frequency

The children’s average cooking self-efficacy (21.8 ± 2.9 out of 24 total points) was high. Children had positive attitudes towards cooking (25.7 ± 4.4 out of 30 total points). The majority reported having cooking experience (83%). Most of the children had cooked with family (94%), while a few reported cooking with friends (39%) (Table 1). The frequency of helping making food for their meals varied, with 50% of the children reporting sometimes (Table 1). About 41% of the children reported that they sometimes went shopping or helped shop for groceries (Table 1).

### 3.3. Qualitative Findings

#### 3.3.1. Cooking Definition

Most participants shared a simple definition of cooking. For example, some children referred to cooking meals at home and not eating out: *“Cooking means when you prepare your own food at home. Like making stuff with your own two hands. Not going out to a restaurant and stuff”* (15A). One child referred to cooking as heating foods, while another child mentioned that cooking was a mixture: *“Having a lot of vegetables and other mixed foods and mix them together. Like a mixture”* (7H). A few participants shared an advanced definition or more nuanced, in-depth understanding of cooking. One expressed the enjoyment of cooking a meal: *“Cooking? To me cooking means like—It’s like making something that you would enjoy eating, something that you would want to eat, like something you make with love”* (10H).

#### 3.3.2. Cooking Confidence and Importance of Cooking

The qualitative data supported the results obtained from the children’s cooking self-efficacy data. When asked, “On a scale of 1–3 (1 = not confident, 2 = not sure, 3 = confident), how confident are you in your cooking skills?” more than half of the children provided a rating of 3, followed by a rating of 2 and 1. Lack of knowledge and skills were a few reasons shared for choosing “not confident”. One child said, *“Like a one. I don’t know exactly know how to cook. I haven’t learned at all, really”* (14H). Similarly, children who selected “not sure” expressed their lack of knowledge, skills, and confidence: *“Because I’m not good at cooking”* (13A). Children who selected “confident” shared that family behavior, exposure, or skill were reasons for their confidence in their cooking skills. For example, family members providing positive feedback on the child’s cooking gave them confidence: *“Because mainly when I cook because it comes out right and my family says they like it very much”* (8A). Children also identified exposure, being around others (e.g., parent, family member) who cooked: *“Because usually when my parents are cooking or when my sister’s cooking, I’m there in the kitchen and helping them make this meal”* (2A). The participants’ belief in his/her cooking skills also gave them confidence: *“Because like even though sometimes I burn stuff or whatever, most of the time it comes out pretty good”* (15A).

When asked, “On a scale of 1–3 (1 = not important, 2 = not sure, 3 = important), how important is it to learn to cook?” the majority of the children provided a rating of 3. Life skills, defined as skills needed for the future, were one of the main reasons shared by the children for selecting a rating of 3. One child shared, *“I say a three because you have to survive eating. I mean cooking like when you grow up you like when you’re an adult you have to know how to cook”* (9A). When discussing life skills, some children spoke about preparing healthy meals at home versus eating fast food: *“I think it’s important to learn how to cook because if you don’t know how to cook, it might be hard for you to eat every day at home. For example, like if you’re in college and you don’t know how to cook, you might want to have to grab fast food and fast food isn’t like the healthiest thing. If you know how to cook, you could probably just cook something healthy for yourself whenever you want to”* (10H). Independence was another reason shared for choosing a rating of 3: “*Because if you don’t know how to cook you can’t make yourself something healthy or make something yourself. You rely on someone else*” (18H).

#### 3.3.3. Cooking Meals with Parents

When asked, “Do you cook meals with your parents?” the majority of children said yes. The qualitative data further support that children have cooking experience with their family, and also support that children found cooking to be a positive experience. When asked, “On a scale of 1–3 (1 = do not like, 2 = do not like or dislike, 3 = like), how much do you like to cook meals with your parents?” more than half of the children shared a rating of 3. Children reported learning how to cook and helping out as reasons for enjoying cooking with their parents. For example, one child shared: *“Because I get to help out and learn. Like I would help and also when I’m helping I would learn how to like make different things”* (16A). Some also saw cooking as an opportunity to bond with their parents and/or family members. One child shared how cooking with her parents was a learning and bonding experience: *“I think it’s fun to kind of just learn how to cook and to be there with your parents while they’re doing it, so it’s kind of like bonding at the same time”* (2A).

A few children reported a rating of 2 for their cooking experience with their parents. One child shared a desire for more independence while cooking as a reason: “*Because sometimes she takes over and I want to be independent and make it myself”* (15A). Another child also shared having a limited role in cooking as a reason: *“…I don’t think you would consider it cooking. I mean it’s helping them with small things that they need. Small tasks in the kitchen”* (14H).

When asked about their role in meal preparation, roles were identified as simple preparation (e.g., chopping/cutting, stirring, putting food in the oven, and seasoning foods), or more advanced, defined as the child being actively involved in meal preparation (e.g., cooking, planning a meal). More than half of the children participated in simple preparation. For example, one child shared his experience with his mother: *“Handing her stuff or mixing stuff up on the stove or putting something in the oven”* (9A). A few children noted having an active role in meal preparation. One child shared his cooking experience with instructions from his mother: *“So she had raw chicken pieces. I helped her cut it up. I unfroze it, I thawed it out, and then she asked me to cut it...So I cut them up…”* (1A). Children also shared their favorite foods/meals to cook with their parents. Examples included spaghetti (a popular choice among the participants), breakfast foods, barbecue, fish, baked chicken, tacos, and mole (a traditional Mexican dish).

#### 3.3.4. Role of Technology in Meal Preparation

Children were asked about the role technology could play when cooking with their parents. Most children reported using technology to locate food demonstration videos and recipes. Some children shared using the Internet to search for cooking videos. One child mentioned: *“Because like I’ll be watching YouTube page Tasty, it’s a YouTube channel called Tasty and they do different types of things. And when I want to try it out, we’ll look up all the ingredients and we’ll see what it takes to do”* (8A). Children also noted using the Internet to find recipes: “*I mean it can. Technology can help like you can use the Internet to look up new healthy recipes”* (14H) or *“Well, technology…the last time I cooked for my mom that was like last week we used the phone for the recipe”* (1A). Alternatively, some children interpreted technology to be using kitchen equipment or appliances such as the microwave, air fryer, oven, blender, and toaster.

#### 3.3.5. Grocery Shopping with Parents

The qualitative data demonstrate a high frequency of children helping with grocery shopping. When asked, “Do you go grocery shopping with your parents?” the majority of children reported that they grocery shopped with their parents. When asked about their role in grocery shopping, responses varied and children reported activities such as picking up items on the grocery list, selecting foods, and participating in a more active way (defined as planning or making the grocery list and deciding on purchases). Picking up items on the grocery list was a common role for the children: *“My role is mostly trying to find things on the list that we need”* (14H). Selecting foods, including favorite foods such as fruit and/or vegetables and snacks, was also a common role for children: *“I tell them, when we are at the fruit and vegetable section: ‘Mommy, I want these fruits, I want you to buy me these fruits’”* (7H). Most children reported that they had the ability to choose some foods for meals or their snacks. Some children also reported playing an active role in grocery shopping. For example, one child shared helping her mother save money on purchases: “*I definitely always help her save money by checking discounts and things on clearance and stuff like that”* (10H). Another child noted participating in weighing items and checking the quality of the food: “*I usually just, I weigh how much of the quantity that they need. And I check the quality of the food if it’s good or if it’s bad”* (18H).

## 4. Discussion

The purpose of this research was to explore the perspectives of minority children living in under-resourced communities about meal preparation and grocery shopping behaviors. Although previous studies have reported quantitative data on children’s cooking self-efficacy and attitudes [24,25,26], this study is distinctive as it presents qualitative interview findings from children’s perspectives about their cooking and grocery shopping behaviors in addition to the quantitative data.

The majority of children in our sample reported having cooking experience. In the Cooking with Kids Program, most children reported having cooking experience [28,29]. Furthermore, most of the children in our study helped with meal preparation on varying frequency levels (i.e., sometimes, often, always). Previous research has found that the majority of fifth graders in Canada (63%) and adolescents in Minnesota (68.6%) helped with meal preparation at least once a week [19,20]. However, another study [24] found that 30% of children helped with meal preparation at least once daily. Children in our sample reported cooking with family more than cooking with friends. Similar to our research, the majority of 9–14-year-olds in southwestern Canada [25] and the majority of children in the Cooking with Kids Program [28,29] reported cooking with family more than cooking with friends. In addition, the majority of children in our sample helped with grocery shopping on varying frequency levels (i.e., sometimes, always, often). Another study found that at least half of the adolescents helped with grocery shopping [20]. These findings support earlier research that children and adolescents are helping with meal preparation and grocery shopping.

The children in the study exhibited high cooking self-efficacy and positive attitudes toward cooking. Previous research with sixth to eighth graders found a positive association between child’s meal preparation frequency and cooking self-efficacy [25]. Moreover, a study from Malaysia with 9–11-year-olds found that positive attitudes, higher self-efficacy towards healthy meal preparation, and higher self-efficacy for making healthy food choices were positively associated with children’s involvement in healthy meal preparation [26]. Findings from cooking and nutrition education programs [27,28,29,31] also confirm that children’s involvement in meal preparation improves their psychosocial factors regarding meal preparation and healthy eating choices.

In our study, the qualitative data further supported the quantitative data on cooking self-efficacy, cooking meals with parents, and grocery shopping with parents. The qualitative data also highlighted children’s perspectives on the importance of learning to cook, their roles in meal preparation and grocery shopping, and the role of technology in meal preparation. The majority of children shared a basic understanding of cooking and provided insight into their cooking confidence level. To our knowledge, these are unique contributions to the literature and can help inform the development of future nutrition and cooking education programs designed for minority children from under-resourced households.

The children in the study reported life skills and independence as reasons for the importance of learning to cook. Similar to our study, Latinx children from low-income families noted that cooking was useful and needed to support future roles (e.g., marriage) [30]. In addition, 6–11-year-old children in New Jersey believed it was important to learn to cook in preparation for adulthood [34]. In our study, 8–13-year-old children shared their personal experience of cooking with their parents and meal preparation roles, including cutting, stirring, putting food in the oven, and seasoning foods. In contrast to our research, the study with 6–11-year-old children in New Jersey reported children participating in fewer meal preparation roles, including setting the table, pouring drinks, washing dishes, and cutting up foods [34]. Age differences must be taken into consideration for the differences observed in meal preparation roles.

In our study, children used technology to locate recipes and cooking demonstration videos online. Comparable to our study, Latinx children also looked online for recipes and for videos and used digital platforms such as Facebook and YouTube [30]. The use of technology is an important tool among children and teens during meal preparation and is needed in future programming. Our study also highlighted children’s personal experiences of grocery shopping with their parents and grocery shopping roles. Picking up items on the grocery list was a common role. Similarly, the study from New Jersey reported that 6–11-year-olds helped pick items from their mother’s grocery list [34]. Furthermore, in our study, children reported selecting foods of their choice, helping write the grocery list, and deciding on purchases. Recognizing the variety of roles children play in meal preparation and grocery shopping is important to the development of future nutrition and cooking education programs.

The strengths of the study included conducting separate semi-structured interviews with children without the involvement of parents, interviewers trained in qualitative methods, and focusing recruitment on both Black/African American and Hispanic families in under-resourced communities. Limitations of this study include the small sample size, conducting the research in a specific geographic region of the United States (a large metropolitan area in Texas), and the inability to connect children’s perspectives on meal preparation and grocery shopping with their dietary outcomes.

## 5. Conclusions

In conclusion, this research highlighted that minority children living in under-resourced communities participate in meal preparation and grocery shopping, understand what cooking is and the importance of learning to cook, use technology in meal preparation, and exhibit high cooking self-efficacy and positive attitudes towards cooking. The qualitative data contributed insightful information into Black/African American and Hispanic children’s experiences with meal preparation and grocery shopping. The role of the child is equally as important as the role of the parents in improving eating behaviors. Children’s perspectives can inform the development of innovative nutrition and cooking education programs and help reduce disparities in diet quality for minority children and families from under-resourced communities.

## Figures and Tables

**Table 1:** Cooking experience and frequency of grocery shopping and food preparation.

	Yes	No
A. Cooking experience	% (*n*)	% (*n*)
Cooking food before	83.3 (15)	16.7 (3)
Cooking food with friends	38.9 (7)	61.1 (11)
Cooked food with family	94.4 (17)	5.56 (1)
B. Grocery shopping and food preparation frequency	*n*	%
Grocery shopping frequency *		
Sometimes	7	41.2
Often	6	35.3
Always	4	23.5
**Family food preparation frequency**		
Never	2	11.1
Sometimes	9	50.0
Often	4	22.2
Always	3	16.7

*: Data missing for one participant.

## Data Availability

The datasets generated and/or analyzed during the current study are not publicly available due to concerns regarding privacy, but select data are available from the corresponding author upon reasonable request.
